# Successful Osteopathic Manipulative Treatment of Foot Drop

**DOI:** 10.7759/cureus.26590

**Published:** 2022-07-05

**Authors:** Leonid Tafler, Victor Katz, Vadim Kolesnikov, Ranjodh Singh

**Affiliations:** 1 Primary Care, Touro College of Osteopathic Medicine, New York, USA; 2 Spine Surgery, New York University (NYU) Langone Hospital Long Island, Brooklyn, USA; 3 Radiology, Sinai Diagnostics, Brooklyn, USA; 4 Medicine, Touro College of Osteopathic Medicine, New York, USA

**Keywords:** chronic lower back pain, lumbar degenerative disorder, sacroiliac joint dysfunctional pain, osteopathic manipulative medicine, drop foot

## Abstract

This case report presents a 63-year-old male patient with chronic left foot drop. The etiology for his condition most likely involved lateral lumbar stenosis and/or sacroiliac joint dysfunction resulting in radiculopathy and subsequent symptoms. The patient was previously recommended a surgical approach for his condition. After an extensive osteopathic examination and application of a high-amplitude low-velocity technique, the patient reported a significant improvement in his pain and resolution of his foot drop. To the best of the author’s knowledge, this is the first reported case of the use of osteopathic medicine in the successful treatment and management of left foot drop most likely secondary lumbar stenosis and/or sacroiliac joint dysfunction. The aim of this case report is to discuss the possible mechanisms by which the condition may have been resolved and the role that osteopathic treatment played in it.

## Introduction

Foot drop is generally defined as the loss of voluntary ankle dorsiflexion [1]. It oftentimes presents with a steppage gait with an audible slap when the foot hits the ground due to weakness in the dorsiflexors of the foot [1,2]. The most common causes of this condition are L5 radiculopathies and peroneal peripheral neuropathy [3-5]. Other causes include anterior horn disease, spinal cord lesions, brain disorders, sciatic nerve compression, lumbar plexopathies, and radiculopathies secondary to herniated nucleus pulposus or foraminal stenosis [4-8].

A database search through PubMed using the search terms “foot drop,” “OMT,” “OMM,” and “Osteopathic treatment,” revealed only one relevant case study concerning the osteopathic treatment of a patient with drop foot secondary to common peroneal nerve compression [9]. To the best of our knowledge, there is no established osteopathic literature detailing the use of osteopathic medicine in the treatment and management of foot drop secondary to lumbar stenosis and sacroiliac joint (SIJ) dysfunction.

We present the case of a 63-year-old male who presented to an osteopathic primary care setting to manage a long-standing foot drop and was successfully treated and managed due to an osteopathic approach to his condition.

## Case presentation

A 63-year-old male patient presented to a primary care office with left foot weakness. The patient’s past medical history consisted of hypertension, right inguinal hernia repair, and appendectomy. The patient reported an active lifestyle involving jogging and skiing in the past.

**Figure 1:**
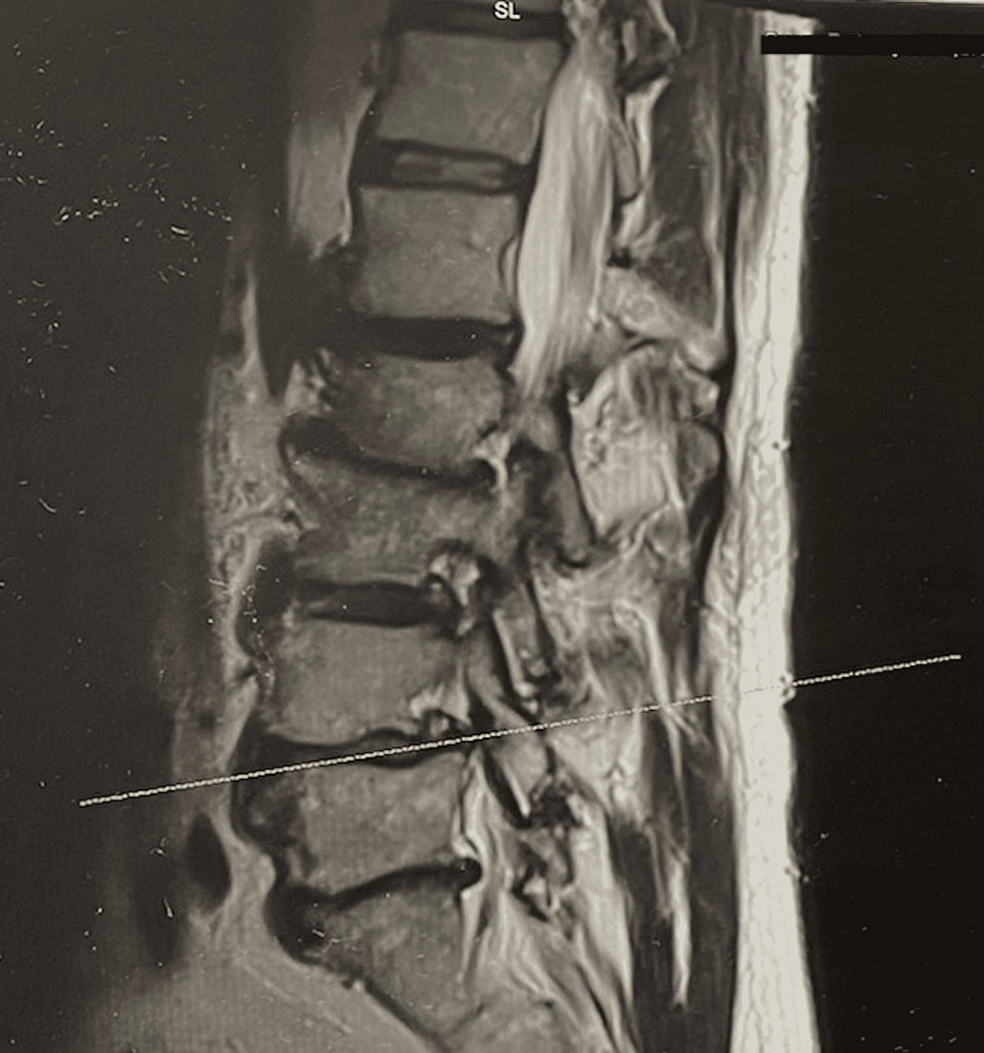
The sagittal MRI image prominently shows severe stenosis at the L4-L5 level, as indicated by the horizontal line, with nerve root impingement as well as L5-S1 foraminal stenosis and nerve root impingement

The patient noticed weakness in his left foot starting more than five years ago but did not visit a health professional at the time due to the minor concerns that it presented. He only received occasional massages and non-steroidal anti-inflammatory medication, as needed. However, the foot weakness gradually worsened, and he also began noticing pain in the lumbar region that radiated down the left leg toward the dorsum of the foot. Eventually, the patient began having difficulties conducting his everyday tasks due to pain that he rated as 9/10 and reported that he could not walk without a constant left foot drop resulting in a limp. The patient subsequently went for a neurosurgical consult. Neurological examination findings showed intact cranial nerves II-XII, motor strength 5/5 throughout the body except for 4/5 in left dorsiflexion, and extensor halluces longus. The sensory exam was intact to touch and proprioception except for decreased sensation in the first three digits of the left foot. There was a notable loss of deep tendon reflexes in the left knee, and the patient was unable to heel walk on the left, although he was successfully able to toe walk. Pertinent MRI findings showed severe left L4-5 lateral recess and foraminal stenosis and left L5-S1 lateral recess and foraminal stenosis (Figure 1). In addition, the L4-5 foraminal stenosis was found to be exaggerated by 1 cm spondylolisthesis of L4 on L5. The patient was diagnosed with left drop secondary to lumbar spinal stenosis. He was offered laminectomy decompression with lumbar fusion at the L4-5 level. The patient then received a second consult for orthopedic surgery. After workup, he was similarly offered a surgical approach for his condition.

Prior to undergoing the proposed procedure, the patient came to our office for an osteopathic approach for his left foot drop. Initial vital signs were stable, and a focused osteopathic exam revealed the following somatic dysfunctions: loss of left foot dorsiflexion, iliac crests of equal height, and the active range of motion of the left hip revealed 4/5 motor strength in flexion and 2/5 muscle strength in left foot dorsiflexion. Left fibula and tibia motion revealed no gross deformities or somatic dysfunctions. Passive range of motion of the left hip, knee, and foot were unremarkable. There was significant hypertrophy of the lumbar paraspinal muscles.

After an extensive osteopathic workup and discussion, the patient elected to have an osteopathic treatment performed. Myofascial and muscle energy techniques were done prior to performing HVLA (high-velocity low-amplitude) on the thoracic, lumbar, and sacral areas with an associated loud audible clicking sound in the lumbosacral region. After the initial treatment, the patient revealed improvement in his pain. The patient then received osteopathic treatment twice a week, and after four sessions in two weeks, the patient reported significant improvement in his left foot drop. Subsequent physical exam findings showed 5/5 left foot dorsiflexion motor strength and 5/5 left hip flexion motor strength. He was also successfully able to heel walk without much difficulty. His sensation in the lower left leg was normal with a still prominent left lumbar hypertrophy. The patient continues to receive treatment once a week and no longer reports left foot drop or lumbar spinal pain. The patient was sent for a post-treatment MRI. The post-treatment revealed no significant difference from the initial imaging.

## Discussion

The lumbar spine consists of five lumbar vertebras from which five respective lumbar nerve roots emerge from the lateral spinal recess [8]. Over time, lumbar degenerative diseases, such as lumbar spinal stenosis and lumbar disc herniation, can occur which lead to lumbar radiculopathies including foot drops. Previous research has shown that drop foot occurs in 0.6%-7.7% of cases that involve lumbar degenerative diseases [7]. More specifically, the L5 nerve root has been found to be the most common region of lumbar radiculopathy in cases involving foot drops [5]. This is most likely due to the osteoarthritic changes that occur at the L4/L5 region, more so than other intervertebral segments, which predispose it to the majority of degenerative spondylolisthesis cases [10,11]. In our case, MRI confirmed the presence of severe left L4-5 lateral recess and foraminal stenosis and 1 cm spondylolisthesis of L4 on L5.

Spinal stenosis refers to the narrowing of the spinal canal. It can be broken down based on anatomical locations in the spines into two general categories: central and lateral [12,13]. Central spinal stenosis refers to degenerative changes involving the area between the facet joints, which comprises the dura mater and its contents [12]. Lateral stenosis occurs in the spinal region that exists in the lateral recess, foraminal, and extraforaminal spaces [12]. Although there is no definitive set of symptoms for this condition, it typically presents with burning or numbness in the lower back and buttocks that can radiate down the affected lower extremity [13]. Spondylolisthesis is the anterior displacement of one vertebral body over another. Oftentimes, this degenerative condition is the result of osteoarthritic changes to the facet joints, typically the lumbar region, which predisposes to L4 forward slippage on L5 [11]. This can further exacerbate impinged L5 nerve roots, particularly in cases with preexisting spinal stenosis. This can result in prominent signs of L5 radiculopathy including loss of foot dorsiflexion due to impaired first toe dorsiflexion and extensor halluces muscle along with sensory loss on the lateral side of the lower leg and dorsal surface of the foot [5,13].

We believe that the one potential mechanism behind the rapid reversal of the foot drop that the patient had been suffering from for years stems from potential alleviation of the L5 nerve root compression secondary to lateral foraminal stenosis and spondylolisthesis. This was accomplished through the osteopathic treatments provided at the time. Initially, myofascial release and muscle energy were performed in the thoracic and lumbar region to loosen the paraspinal muscles and prep for HVLA. Subsequently, the patient was positioned for mid-thoracic HVLA and lower-thoracic HVLA [13]. After doing so, we decided to treat the lumbar region of the spine with HVLA except a non-traditional path was chosen as positioning was like that of the lower-thoracic setup [13]. The purpose of doing so was under the assumption that the 1-cm spondylolisthesis noted in the MRI at the L4-L5 region may have served as a movable “space” due to increased laxity, which was aggravating the compressed L5 nerve root. In performing HVLA in this region, we may have slightly shifted the positioning of this movable “space” just enough, which removed excess pressure on the root and released it such that it alleviated the patient’s symptoms. Performing subsequent weekly therapy ensured that the spondylolisthesis did not reapply excess pressure on the nerve root as it was adjusted on a consistent basis. Furthermore, the patient was also advised to place a pillow in his lower lumbar region below the spondylolisthesis so that it increased the lordosis and decreased the misalignment of L4 on L5 further decompressing the impinged nerve roots. Of note, the pre- and post-MRI review did not reveal any significant improvements in stenosis or spondylolisthesis, which make it difficult to assess the validity of this possible mechanism. A confounding factor was that the two MRIs were performed with different machines at two different facilities, which made the comparison of the two studies more difficult.

Another possibility is that the radicular symptoms are secondary to an SIJ dysfunction. SIJ dysfunction has been associated with lower extremity radicular symptoms that may be due to regional lumbosacral nerves in contact with SIJ [14]. The L5 nerve root runs over the SIJ anteriorly, predisposing it to irritation or stretch if the SIJ is out of position or subluxed. Unfortunately, it is difficult to assess the SIJ dysfunction as no physical exam finding is reliable or validated although the gold standard involves intra-articular blockage for alleviation of SIJ-related pain [14]. In our case, the physical exam done did not provide any significant findings in regard to the SIJ. In addition, imaging was not performed specifically to assess the SIJ, which could have shed further light on the matter. Nonetheless, we hypothesize that the patient may have had a potential SIJ dysfunction that was irritating the surrounding lumbosacral nerves resulting in his symptoms. Subsequent osteopathic manipulation in the lumbosacral region may have alleviated this subluxation resulting in the rather quick and steady relief of his radicular symptoms.

## Conclusions

This case report presents the successful treatment of a patient with chronic left foot drop using osteopathic techniques. The patient has continued his treatment regimen and at-home treatment and has reported no relapse of his initial complaint. To the authors’ knowledge, this is the first case report demonstrating the successful management of foot drop most likely secondary to lumbar stenosis, spondylolisthesis, and SIJ dysfunction. Although surgical intervention may be warranted in such cases, other diagnoses of foot drop and radiculopathy should still be considered including the diagnosis of SI joint pathology. We hope that other physicians and healthcare professionals can utilize the findings of this case report to further examine and expand on the utility of osteopathic treatment for patients suffering from similar medical conditions.

## References

[1] Agarwal P, Zehnder RJ (2014). Foot drop. Neurologic Differential Diagnosis: A Case-Based Approach.

[2] (2021). Causes and evaluation of neurologic gait disorders in older adults. https://www.uptodate.com/contents/causes-and-evaluation-of-neurologic-gait-disorders-in-older-adults?search=steppage%20gait&source=search_result&selectedTitle=2~150&usage_type=default&display_rank=2.

[3] Stewart JD (2008). Foot drop: where, why and what to do?. Pract Neurol.

[4] Ma J, He Y, Wang A, Wang W, Xi Y, Yu J, Ye X (2018). Risk factors analysis for foot drop associated with lumbar disc herniation: an analysis of 236 patients. World Neurosurg.

[5] Carolus AE, Becker M, Cuny J, Smektala R, Schmieder K, Brenke C (2019). The interdisciplinary management of foot drop. Dtsch Arztebl Int.

[6] Westhout FD, Paré LS, Linskey ME (2007). Central causes of foot drop: rare and underappreciated differential diagnoses. J Spinal Cord Med.

[7] Nakashima H, Ishikawa Y, Kanemura T (2020). Neurological function following early versus delayed decompression surgery for drop foot caused by lumbar degenerative diseases. J Clin Neurosci.

[8] Nori LS, Stretanski MF (2021). Foot Drop. https://www.ncbi.nlm.nih.gov/books/NBK554393/.

[9] Lavelle JM, McKeigue ME (2009). Musculoskeletal dysfunction and drop foot: diagnosis and management using osteopathic manipulative medicine. J Am Osteopath Assoc.

[10] Savarese RG, Capobianco JD, Cox JJ (2009). OMT Review: A Comprehensive Review in Osteopathic Medicine. United States.

[11] Tamrakar BB, Tandra N, Yonghui H, Dapeng L, Jifu S (2014). Radiological evaluation of lumbar instability. IOSR J Dent Med Sci.

[12] Lee SY, Kim TH, Oh JK, Lee SJ, Park MS (2015). Lumbar stenosis: a recent update by review of literature. Asian Spine J.

[13] DiGiovanna EL, Schiowitz S, Dowling DJ (2004). An Osteopathic Approach to Diagnosis and Treatment.

[14] Buijs E, Visser L, Groen G (2007). Sciatica and the sacroiliac joint: a forgotten concept. Br J Anaesth.

